# A high-throughput, plate reader-based method for the assessment of oxidative stress in suspension mammalian cells using CellROX Green

**DOI:** 10.1007/s00418-026-02517-2

**Published:** 2026-07-27

**Authors:** Miyah N. Awad, Amanda N. Abraham, Philipp Reineck, Sara Pourshahrestani, Izabela Milogrodzka, Aaron Elbourne, Tamar L. Greaves, Gary Bryant, Saffron J. Bryant

**Affiliations:** School of Science, STEM College, RMIT University, Melbourne, VIC 3001 Australia

**Keywords:** Reactive oxygen species (ROS), Microplate fluorometry, High-throughput screening, Oxidative stress probe, Suspension cells, Plate reader protocol

## Abstract

The measurement of intracellular reactive oxygen species (ROS) levels provides critical insights into cell health and pathophysiology. Here, we present a semi-quantitative, plate reader-based protocol using CellROX Green that can be used for the real-time measurement of general ROS levels of viable suspension mammalian cells. With menadione being the oxidative stress inducer, a cell concentration of 100,000 cells per well, a CellROX Green concentration of 10 µM, and a CellROX Green incubation time of 1 h was shown to provide the most accurate measurement of intracellular ROS levels in viable human monocyte leukemia (THP-1) cells. Unlike plate reader protocols using other general ROS-sensitive fluorogenic probes, this plate reader protocol eliminates the washing step of the unbound probe prior to measuring fluorescence, making it more suitable for suspension cells. Using the presented protocol, the fluorescence of up to 96 samples can be measured in under 1 min, making it ideal for rapid screening applications where moderate to large differences in oxidative stress levels are to be measured.

**Supplementary Information:**

The online version contains supplementary material available at 10.1007/s00418-026-02517-2.

## Introduction

Reactive oxygen species (ROS) are oxygen-containing molecules that are highly reactive due to the presence of unpaired electrons, which enables their ability to readily form radicals or strong oxidants (Cao et al. 2022; Liu et al. 2021). Under normal conditions, intracellular ROS have a pivotal role in regulating cell differentiation, metabolism, and growth (Agarwal et al. 2022; Armstrong and Whiteman 2007; Liu et al. 2021). However, under stressful conditions, ROS production can significantly increase and overwhelm cells’ endogenous antioxidant mechanisms (Ariyan et al. 2021; Mateo-Otero et al. 2021; Ruijter et al. 2024). Oxidative stress, resulting from the accumulation of intracellular ROS, can lead to altered cell signalling, degradation of cellular components (Armstrong and Whiteman 2007), cellular senescence (Len et al. 2019), and ultimately cell death (Len et al. 2019). Thus, measurements of intracellular ROS levels provide insights into cell health and pathophysiology (Chung and Duchen 2022).

The quantification of intracellular ROS is particularly challenging due to their high reactivity, short half-lives, and relatively low abundance in cells (Fan and Li 2014; Figueroa et al. 2018; Murphy et al. 2022). ROS-detecting fluorogenic probes often possess high sensitivity, making them a popular tool for measuring intracellular ROS levels (Gomes et al. 2005). In a reduced state, they exhibit little to no fluorescence (Griendling et al. 2016), but upon oxidation by ROS they elicit a strong fluorescent signal which can be captured using techniques such as fluorescence microscopy, flow cytometry, and, in some cases, plate reader-based fluorimetry (Griendling et al. 2016; Kauffman et al. 2016). Plate reader-based fluorimetry uses a benchtop instrument to measure fluorescence emitted by a sample prepared in a well plate. Compared to traditional fluorescence microscopy and flow cytometry, plate reader-based fluorimetry, when paired with a ROS-detecting fluorogenic probe, is considered one of the easiest, fastest, most scalable, accessible, and user-friendly methods to assess oxidative stress in cells (Armstrong and Whiteman 2007; Ng and Ooi 2021).

Most ROS-detecting fluorogenic probes have been designed to detect a single ROS type and/or are specific to a particular ROS location of production. For instance, dihydroethidium fluoresces when oxidised by cytosolic superoxide (Chung and Duchen 2022), MitoSOX Green fluoresces when oxidised by mitochondrial superoxide (Park and Kang 2025), and Amplex Red fluoresces when oxidised by hydrogen peroxide in the presence of peroxidase (Armstrong and Whiteman 2007). Fluorogenic probes designed to non-specifically detect ROS are less common. The membrane-permeable probes dichlorodihydrofluorescein diacetate (DCFDA) (a.k.a. DCFH-DA, CM-H_2_DCFDA, H_2_DCF, H_2_DCFDA) and dihydrorhodamine (DHR123) are the most popular probes used to measure general levels of ROS within cells in vitro (Boulton et al. 2011; Djiadeu et al. 2017; Fan and Li 2014; Kalyanaraman et al. 2012; Ortega-Villasante et al. 2018; Ruijter et al. 2024). Through a two-step process involving the formation of an intermediate radical, the DCFDA and DHR123 probes are oxidized by free radicals to produce the fluorescent dichlorofluorescein (DCF) and rhodamine (RhH_2_) compounds, respectively (Kalyanaraman et al. 2012; Wardman 2007). These fluorescent products can be measured using plate reader-based fluorometry, flow cytometry, and fluorescence microscopy (Djiadeu et al. 2017; Invitrogen 2006).

Despite their popularity, the DCFDA and DHR123 probes share several key drawbacks. Firstly, both probes are susceptible to self-amplification of the fluorescent signal via a redox-cycling mechanism involving intermediate radicals (DCF^•−^ and DHR^•^) (Armstrong and Whiteman 2007; Kalyanaraman et al. 2012; Ofosu et al. 2023; Ortega-Villasante et al. 2018; Ruijter et al. 2024; Zhang et al. 2024). DCFH can also be oxidised by cytochrome *c* (Dikalov and Harrison 2014), heme peroxidases (Dikalov and Harrison 2014), and other non-radical species, including transition and redox-active metals such as Fe^2+^(Granato 2023), again resulting in an artificial increase in fluorescence. Secondly, both probes are extremely air and light sensitive, with the DCFDA probe requiring storage under dry argon or nitrogen (Invitrogen 2006). Finally, when using plate reader-based fluorometry to measure fluorescence, washing of the samples after incubation with the probes is required before the fluorescence signal is measured (Bioquochem 2023; Ruijter et al. 2024). It has been found that the DCFDA probe and the intermediate product DCFH can translocate into the supernatant and interact with exposure treatments and extracellular ROS to increase fluorescence (Ruijter et al. 2024). Washing can lead to cell loss (Ruijter et al. 2024), which can be problematic for suspension cell lines with low cell concentrations. With the limitations of the DCFDA and DHR123 probes well recognised in the literature (Dikalov and Harrison 2014; Kalyanaraman et al. 2012; Ruijter et al. 2024; Yazdani 2015; Zhang et al. 2024), recent studies have opted for a different ROS-sensitive fluorescent probe as a general oxidative stress indicator—CellROX Green.

CellROX Green is a cell-permeable fluorogenic probe which can be used for generalised intracellular radical detection (Invitrogen 2012; Ortega-Villasante et al. 2018; Zhang et al. 2024). In a reduced state, CellROX Green is weakly fluorescent (Invitrogen 2012). However, upon oxidation by intracellular ROS, it binds to DNA and exhibits a strong fluorogenic signal localised primarily around the DNA in the nucleus and mitochondria (Chittiboyina et al. 2018; Invitrogen 2012; Zhang and Bar-Peled 2023). Though the exact chemical structure of CellROX Green has not been revealed by its manufacturers, the spectral information suggests that the probe belongs to the DCF-like family of fluorophores (Ortega-Villasante et al. 2018). The one-step mechanism of CellROX Green makes it advantageous over the DCFDA and DHR123 probes as it requires no further processing (Ortega-Villasante et al. 2018). Other advantages identified by the manufacturer include its increased sensitivity, stability, simplicity, easy storage conditions, and its fixability (Invitrogen 2012).

In the literature, the fluorescence of CellROX Green has been measured using traditional fluorescence microscopy (Carpentieri et al. 2022; Wang et al. 2014; Yokoyama et al. 2017) and flow cytometry (Bradley et al. 2020; Deragon et al. 2020; Kelesidis et al. 2020). However, these methods can be relatively labour-intensive and time-consuming which make them non-ideal for large-scale screening applications. CellROX Green should be compatible with plate reader-based fluorimetry (Invitrogen 2012), yet a plate reader-based protocol has not been reported to date. There are a couple of reports of related plate reader-based measurements: McBee et al. (2017) report fluorescence measurements of bacteria stained with CellROX Green using a microplate reader; and Zwicker et al. (2022) report a method in which the lysates of adherent human monocyte leukemia (THP-1) cells stained with CellROX Green were used to measure cellular ROS via plate reader-based fluorometry. However, to the best of our knowledge, no published protocol exists which uses CellROX Green with plate reader-based fluorometry to directly measure ROS within living and intact mammalian cells.

In this paper, we present a semi-quantitative plate reader-based protocol using CellROX Green that can be used to measure intracellular ROS levels in viable THP-1 cells in a 96-well plate. Several protocol parameters including cell concentration, CellROX concentration, incubation time with CellROX Green, excitation and emission wavelengths, and emission bandwidth were optimised for the measurement of ROS in menadione-treated THP-1 cells. This protocol has the potential to be used with other suspended mammalian cell lines. The plate reader protocol is most suited to measure moderate to large differences in oxidative stress levels and thus could have widespread use in rapid screening applications. For instance, this protocol could be used to screen for novel anticancer or anti-inflammatory drugs by measuring the ROS levels of cells after treatment, or could be used to screen for new antioxidants for cryopreservation applications by measuring the post-thaw ROS levels of cryopreserved mammalian cells.

## Materials and methods

### Model cell lines

Human monocyte leukemia (THP-1) cells were used as a representative suspension mammalian cell line and were purchased from ATCC (American Type Culture Collection, MASASSAS, VA). Human immortalized keratinocyte (HaCaT) cells were used as a representative adherent mammalian cell line and were provided by collaborators from RMIT University (Melbourne, Australia). Following the ATCC standard protocol for cell survival, frozen cells were thawed at 37 °C and diluted in 5 mL of fresh media containing 1% penicillin/streptomycin (catalog no. 15070063, Gibco, Thermo Fisher Scientific, VIC, AUS) and 10% foetal bovine serum (FBS) (code: SFBS-AU, Bovogen, VIC, AUS). The diluted suspension of cells then underwent centrifugation at 200 × *g* for 5 min, the supernatant was removed, and the pellet of cells was re-suspended in fresh media. The cells were cultured at 37 °C in a humidified environment containing 5% CO_2_. Methods used for cell maintenance have been described in detail elsewhere (Awad et al. 2024). Briefly, every 2 to 3 days, the THP-1 and HaCaT cells were refreshed with fresh Roswell Park Memorial Institute (RPMI) medium (catalog no. 11835030, RPMI1640, Gibco, Thermo Fisher Scientific, VIC, AUS) or Dulbecco’s modified Eagle’s medium (DMEM) (catalog no. 21063029, Gibco, Thermo Fisher Scientific, VIC, AUS), respectively. THP-1 and HaCaT cells were subcultured when cell density surpassed 8 × 10^5^ cells/mL (THP-1) or confluency reached 70–80% (HaCat). The trypan blue exclusion test was used to determine cell number and viability, as the dye can only penetrate and stain cells with damaged cell membranes (Awad et al. 2024). Automated cell counting was facilitated by the Countess III Cell Counter (Thermo Fisher, VIC, AUS), with cell viability calculated using the following equation:

1 $$\mathrm{Viability} \left(\mathrm{\%}\right)=\frac{\text{Live cell number}}{\text{Live cell number} + \text{Dead cell number}} \times 100$$

### Protocol development with THP-1 cells

**Fig. 1:**
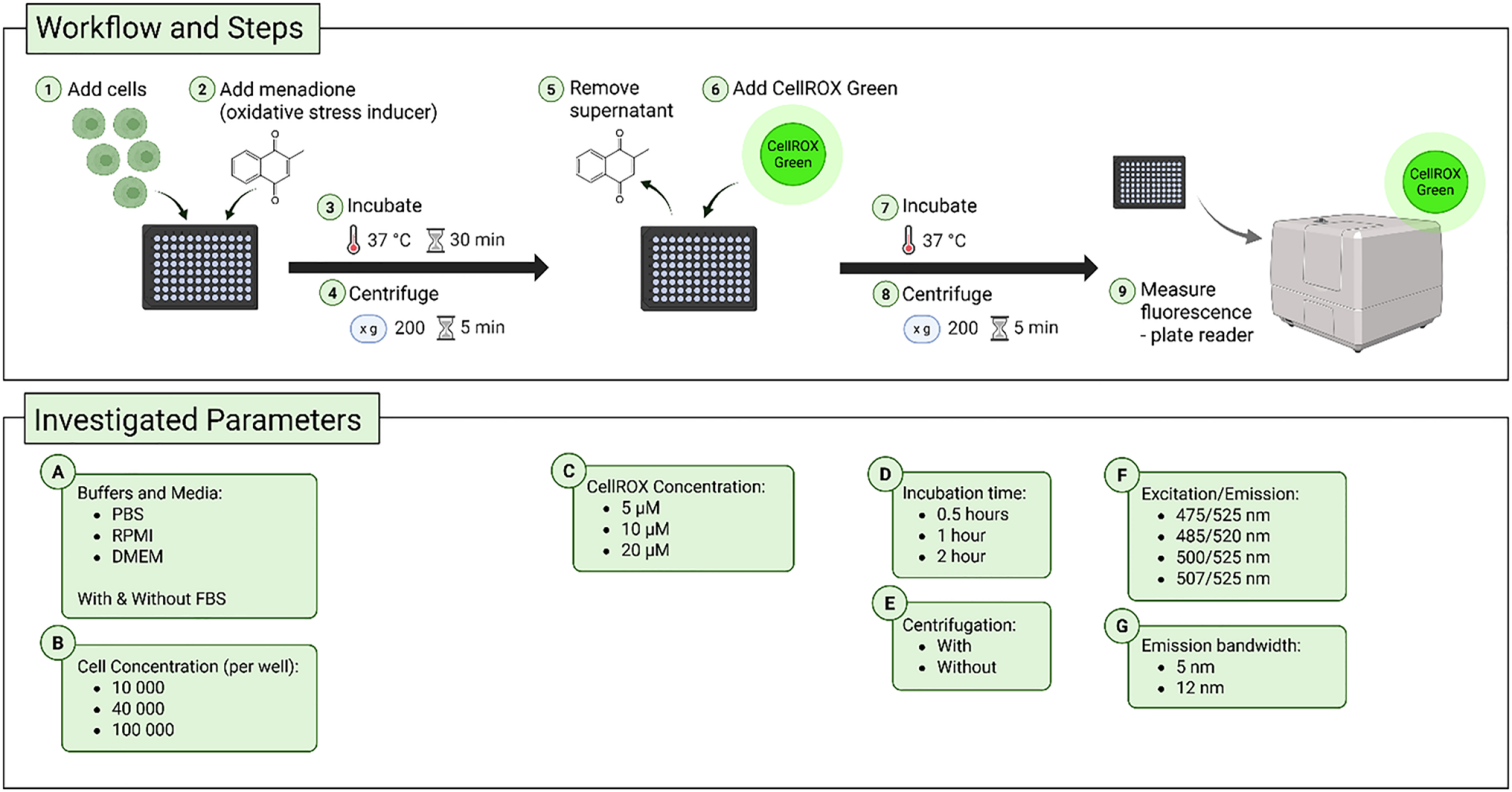
Schematic of basic protocol used to develop a plate reader-based assay using CellROX Green to measure intracellular ROS levels of human monocyte leukemia (THP-1) cells. Each step is represented by a number (1–9). Investigated parameters are listed at the bottom of the diagram, directly below the relevant workflow step. Created with BioRender.com. Phosphate buffer saline (PBS), RPMI; Roswell Park Memorial Institute medium (RPMI), Dulbecco’s modified Eagle’s medium (DMEM), foetal bovine serum (FBS)

Figure 1 illustrates the basic protocol for measuring intracellular ROS levels in THP-1 cells using CellROX Green, with the parameters optimised in this work shown in the green boxes. In a 96-well black clear-bottom plate (catalog no. 165305, Thermo Fisher Scientific, NY, USA), 95 µL of THP-1 cells was added to at least triplicate wells. The buffers and media used included phosphate buffer saline (PBS), RPMI, and DMEM (for HaCaT cells), supplemented with and without FBS (parameter A). Three cell concentrations were used: 10,000 cells/well, 40,000 cells/well, and 100,000 cells/well (parameter B).

To induce oxidative stress, 95 µL suspensions of cells were incubated at 37 °C for 30 min with menadione (catalog no. M5625, ≥ 98.0%, Sigma-Aldrich, VIC, AUS) diluted in 5 µL of dimethyl sulfoxide (DMSO) (catalog no. D8418, ≥ 99.9%, Sigma-Aldrich, VIC, AUS) to give final concentrations of between 1 µM and 100 µM of menadione depending on the experiment. Menadione is an aromatic ketone which can generate intracellular ROS at multiple sites within cells (Loor et al. 2010). It is often used as an oxidative stress inducer in antioxidant studies and therefore was chosen for this study. Toxicity testing showed that treatment of THP-1 cells with 100 µM of menadione for 30 min at 37 °C resulted in no significant cell death after 3 h compared to the untreated control (Fig. S1). Therefore, a 30-min incubation with 100 µM of menadione was deemed appropriate to trigger oxidative stress.

After incubation, the plate was centrifuged at 200 × *g* for 5 min to pellet the cells. To ensure that the pellet was not disturbed and to minimise cell loss, only 50 µL of the supernatant was removed. CellROX Green (Product number C10444, Thermo Fisher, USA) diluted in PBS was added to each well to give a final volume of 70 µL. Three CellROX Green concentration were tested: 5 µM, 10 µM, and 20 µM (parameter C). The samples were further incubated at 37 °C for either 0.5, 1, or 2 h (parameter D). To determine the necessity of pelleting the cells before obtaining fluorescence, fluorescence was measured before and after centrifugation at 200 × *g* for 5 min (parameter E). The Varioskan Lux (Thermo Fisher Scientific, VIC, AUS) microplate reader was used to collect fluorescence from the bottom of the well plate.

To determine the optimal fluorescence excitation and emission conditions, four excitation and emission wavelength combinations (475/525 nm, 485/520 nm, 500/525 nm, 507/525 nm (parameter F)) and two emission bandwidths (5 nm and 12 nm (parameter G)) were used. According to the manufacturer, the recommended excitation and emission wavelengths for CellROX Green are 485 nm and 520 nm, respectively, with fluorescence microscopy (Invitrogen 2012), and 508 nm and 525 nm, respectively, with flow cytometry (Invitrogen 2014). The first of these conditions was used in this study. However, the second could not be reproduced as the microplate reader requires a minimum difference between the excitation and emission wavelengths of 18 nm with a 5 nm bandwidth and 25 nm with a 12 nm bandwidth. Therefore, an excitation and emission combination of 507 nm and 525 nm, respectively, was examined using an emission bandwidth of 5 nm to most closely reproduce the flow cytometry methodology.

For each experiment, a single fluorescence measurement was obtained for each of at least three wells (i.e. three experimental replicates), and each experiment was conducted at least two times (i.e. two biological replicates). Fluorescence was reported as either an emission spectrum across 500–600 nm, or at a single emission wavelength. For experiments in which normalised fluorescence is reported, the average fluorescence of the untreated cell control was subtracted from the fluorescence of the treated samples and then normalised to the average fluorescence of the untreated control using the following formula:

2 $$\text{Normalised fluorescence}=\frac{\mathrm{T}\mathrm{r}\mathrm{e}\mathrm{a}\mathrm{t}\mathrm{e}\mathrm{d} - \mathrm{U}\mathrm{n}\mathrm{t}\mathrm{r}\mathrm{e}\mathrm{a}\mathrm{t}\mathrm{e}\mathrm{d} }{\mathrm{U}\mathrm{n}\mathrm{t}\mathrm{r}\mathrm{e}\mathrm{a}\mathrm{t}\mathrm{e}\mathrm{d}}$$

Table 1 lists the parameters and other conditions used to optimise the developed protocol. Each experiment featured optimisation of a single parameter and all other parameters remained consistent with the basic protocol as listed in Table 1.

**Table 1:** Protocol parameters investigated as identified in Fig. 1

Parameter ID in Fig. 1	Parameter	Trialled conditions	Basic protocol conditions
A	Buffers and media	PBS PBS + FBS RPMI RPMI + FBS DMEM DMEM + FBS	PBS
B	Cell concentration	10,000 cells/well 40,000 cells/well 100,000 cells/well	100,000 cells/well
C	CellROX Green concentration	5 µM 10 µM 20 µM	10 µM
D	Incubation time	0.5 h 1 h 2 h	1 h
E	Centrifugation	With Without	With
F	Excitation/emission wavelengths	475/525 nm 485/520 nm 500/525 nm 507/525 nm	475/525 nm
G	Emission bandwidths	5 nm 12 nm	12 nm

Apart from the parameter being optimised, the basic protocol conditions were used for all experiments

Phosphate buffer saline (PBS), Roswell Park Memorial Institute medium (RPMI), Dulbecco’s modified Eagle’s medium (DMEM), foetal bovine serum (FBS)

### Flow cytometry

The more sensitive flow cytometry method was used as a practical benchmark for the developed plate reader protocol. In 1.5 mL tubes, 100,000 THP-1 cells were suspended in 380 µL of PBS. This was then incubated with 20 µL of a DMSO solution containing menadione to yield final menadione concentrations of 0 µM, 1 µM, 10 µM, or 100 µM. The samples were incubated at 37 °C for 30 min, then centrifuged at 200 × *g* for 5 min to pellet the cells. Without disturbing the pellet, 350 µL of the supernatant was removed, and the pellet was resuspended in 450 µL of PBS containing CellROX Green to achieve a final concentration of 1 µM of CellROX Green. The samples were further incubated for 1 h at 37 °C per the manufacturer’s protocol (Invitrogen 2014).

The flow cytometry data was acquired using the BD Accuri C6 flow cytometer (Becton, Dickinson and Company, USA). As a result of instrument limitations regarding preset excitation/emission bandwidths, samples were excited at 488 nm, and fluorescence was measured at an emission of 533 nm with a 30-nm bandwidth. This was the closest option to the manufacturer’s recommended protocol available (Invitrogen 2014). The average fluorescence of the samples was calculated from 10,000 events (cells) within the gated region (Fig. S2). The normalised fluorescence was determined using Eq. 2.

### Validating the plate reader-based protocol with suspended HaCaT cells

The plate reader protocol was also tested with suspended HaCaT cells treated with up to 100 µM of menadione. This was done to validate the developed protocol with a second cell line, demonstrating the suitability of the protocol to other cell lines beyond THP-1 cells.

### Statistics

Statistical analysis was conducted where appropriate using GraphPad Prism (version 10.04.1). Values that differed by more than a factor of two from the mean of the other experimental replicates were treated as outliers and excluded from the dataset. Statistical significance was determined using two-way analysis of variance (ANOVA) followed by Tukey’s post hoc test for multiple comparisons. A *p* value ≤ 0.05 was considered statistically significant.

## Results and discussion

### Determining optimal cell and CellROX Green concentrations

**Fig. 2:**
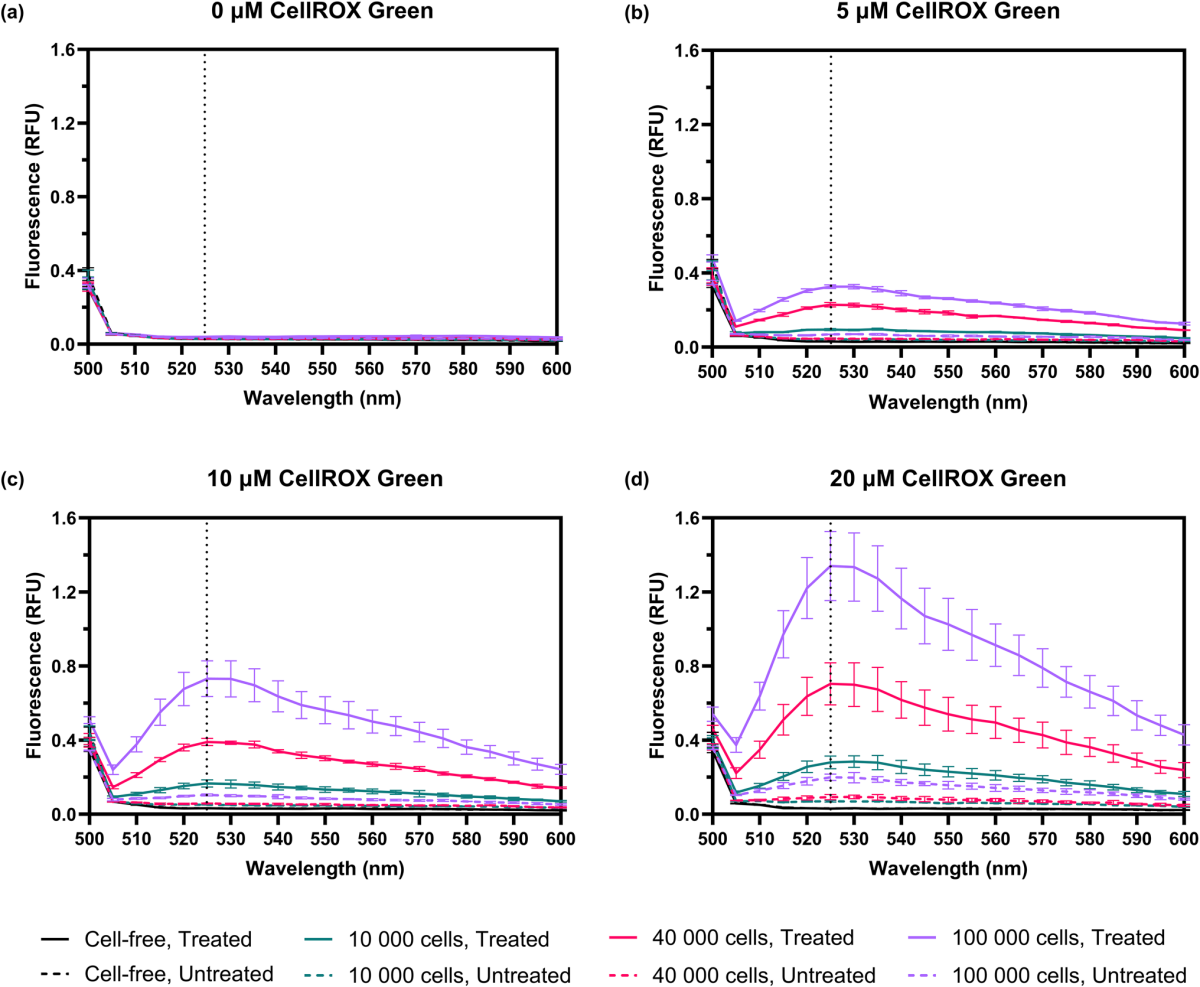
Fluorescence of THP-1 cells incubated with **a** 0 µM, **b** 5 µM, **c** 10 µM, or **d** 20 µM of CellROX Green for 1 h following 30 min of incubation with and without 100 µM of menadione. Samples were excited at a wavelength of 475 nm. The dotted line shows peak emission at 525 nm. Error bars are based on the standard deviation of three experimental replicates

To develop a plate reader-based protocol using CellROX Green for the measurement of intracellular ROS levels in viable suspension cells, several protocol parameters were optimised using THP-1 cells as the model suspension cell line. First, cell concentration and CellROX Green concentration were optimised. THP-1 cells were treated with 100 µM of menadione for 30 min or left untreated in PBS. Toxicity testing showed that treatment with 100 µM of menadione for 30 min did not result in significant cell death after 3 h (Fig. S1) and thus was deemed an appropriate treatment. Figure 2 shows the fluorescence intensity of samples after incubation with different concentrations of CellROX Green for 1 h. As shown in Fig. 2a, unstained samples showed no significant fluorescence, highlighting that neither the cells themselves nor potential residual menadione in DMSO contributed to fluorescence at the emission wavelengths of interest. Across all CellROX Green concentrations, the cell-free samples showed similar fluorescence to the unstained controls. This shows that unbound CellROX Green and menadione do not significantly contribute to background fluorescence. Importantly, this indicates that the samples do not need to be washed to remove unbound CellROX Green before fluorescence data is obtained. This is a significant advantage over the DCFDA and DHR123 probes (Abcam 2026; Bioquochem 2023).

**Fig. 3:**
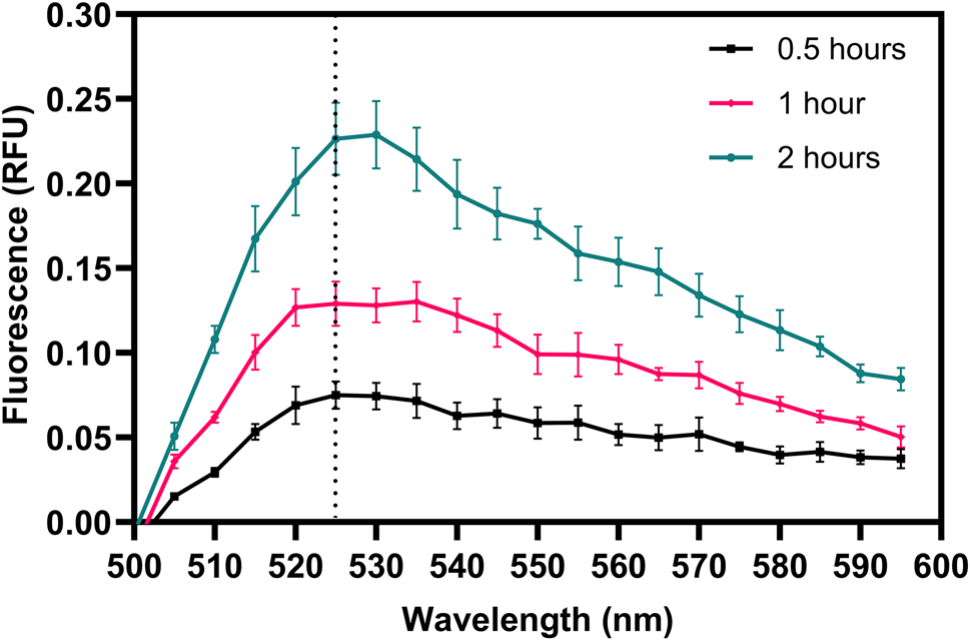
Fluorescence of THP-1 cells incubated for 0.5, 1, or 2 h with 10 µM of CellROX Green following 30 min of incubation with 100 µM menadione. Samples were excited at a wavelength of 475 nm. The dotted line shows peak emission at 525 nm. Error bars are based on the standard deviation of three experimental replicates

Of the treated samples (incubated with 100 µM of menadione), only three samples showed a distinct emission peak at 525 nm (with RFU above 0.5): (1) 100,000 cells, 10 µM CellROX Green (Fig. 2c), (2) 40,000 cells, 20 µM CellROX Green (Fig. 2d), and (3) 100,000 cells, 20 µM CellROX Green (Fig. 2d). Given the high cost of CellROX Green, a cell concentration of 100,000 cells per well paired with a CellROX Green concentration of 10 µM was selected for the developed protocol.

A CellROX Green concentration of 10 µM per sample is comparable to concentrations commonly used for plate reader-based assays with the DCFDA (10–50 µM) and DHR123 (5–25 µM) probes (Abcam 2026; Azzouz and Palaniyar 2023; Bioquochem 2023; Djiadeu et al. 2017). While a cell concentration of 100,000 cells per sample is considered enough cellular material for sufficient fluorescence detection for the DCFDA and DHR123 assays, a cell concentration of 150,000 cells per sample is recommended to account for inevitable cell loss due to the washing step of these protocols (Abcam 2026; Bioquochem 2023). The elimination of the washing step in this CellROX Green protocol is a clear advantage over the DCFDA and DHR123 probes as it allows for a lower cell concentration per sample.

### Determining incubation time with CellROX Green

The manufacturer’s protocol recommends an incubation time of 0.5 h with CellROX Green, up to a maximum of 2 h (Invitrogen 2012). Thus, cells were treated with 100 µM of menadione, and the resulting fluorescence was measured after 0.5, 1 and 2 h of incubation with 10 µM of CellROX Green. Figure 3 shows the average fluorescence of the samples after subtracting the average fluorescence of the untreated samples. As expected, the emission increased with incubation time, with the peak at 525 nm increasing from 0.075 to 0.136 to 0.226 RFU, respectively. However, an extended incubation time of 2 h may increase susceptibility to artefacts such as auto-oxidation, probe-induced toxicity, and signal saturation which may lead to inaccurate results (Jiang et al. 2018; Karges 2026). To avoid this, a 1-h incubation period was identified as the most appropriate for this protocol. This is comparable to the incubation times used with the DCFDA and DHR123 probes (Bioquochem 2023; Invitrogen 2006).

### Measuring fluorescence of different media and buffers with and without cells

**Fig. 4:**
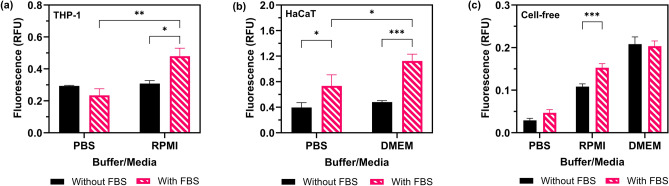
Fluorescence of **a** THP-1 cells, **b** HaCaT cells, and **c** cell-free controls in either PBS or media (RPMI for THP-1, DMEM for HaCaT), with and without FBS supplementation, treated with 100 µM of menadione for 30 min. For **a** and **b**, fluorescence is reported after the fluorescence of the untreated cell controls was subtracted. For **c** raw fluorescence is reported. Fluorescence was captured for all samples at an excitation and emission of 475 nm and 525 nm, respectively. Error bars are based on the standard deviation of three experimental replicates, with a single outlier removed from the following datasets, **b** PBS–Without FBS, RPMI–Without FBS, **c** PBS–With FBS. Notable differences as determined by Tukey’s post hoc testing are shown as follows: *p ≤ 0.05, **p ≤ 0.01, ***p ≤ 0.001

In the previous experiments, PBS was chosen as the buffer as it is much simpler in composition than cell media. However, cell media supplemented with FBS is usually used for in vitro cell experiments. FBS provides hormones, growth factors and other complex biomolecules important for cell survival, growth and proliferation (Liu et al. 2023). To determine if phenol-free cell media supplemented with FBS (complete media) is suitable for the developed protocol, THP-1 and HaCaT cells were treated with 100 µM of menadione in either PBS or media (RPMI for THP-1 cells, DMEM for HaCaT cells), with and without FBS, and the fluorescence was measured at an excitation of 475 nm. Figure 4 shows the fluorescence of the samples at an emission wavelength of 525 nm (see Fig. S3 for the fluorescence spectra). Two media types were used as they are known to have varied compositions (e.g. RPMI contains lower levels of most vitamins and amino acids as compared to DMEM) which may result in different interactions with the CellROX Green (Gong et al. 2023; Price 2017).

For the THP-1 samples, the PBS and RPMI cell samples which were not supplemented with FBS showed statistically similar fluorescence (approximately 0.3 RFU), with relatively little variance across the replicates. However, with the addition of FBS, fluorescence of the RPMI sample increased to 0.5 RFU. The addition of FBS to the PBS sample resulted in a minimal decrease in fluorescence, though this was shown to be statistically insignificant. For the HaCaT cells, the PBS and DMEM samples exhibited statistically similar fluorescence within errors (0.4 RFU and 0.5 RFU, respectively). However, with the addition of FBS, fluorescence of both the PBS and DMEM samples increased to 0.7 RFU and 1.1 RFU, respectively. For both cell lines, larger error bars were seen with samples supplemented with FBS as compared to those without FBS, indicating that the addition of FBS results in greater variance between replicates. While the RPMI cell-free controls with and without FBS showed statistically significant differences in fluorescence, the DMEM cell-free controls with and without FBS showed statistically similar fluorescence. Additionally, the changes in fluorescence observed with the addition of FBS were not consistent across the different buffer and media types.

Taken together, the results highlight that media conditions should be validated empirically by users before routine use of the developed protocol. Supplementation with FBS had an inconsistent effect on fluorescence measurements, indicating that FBS-free media/buffer conditions may be more appropriate for the developed protocol. This may be considered a disadvantage of using CellROX Green as compared to the DCFDA probe which is compatible with FBS-supplemented media according to the plate reader-based protocol (Abcam 2026). Nonetheless, PBS is suitable for most cell types and therefore it is recommended that PBS be used for protocol optimisation.

### Optimising fluorescence excitation and emission conditions

**Fig. 5:**
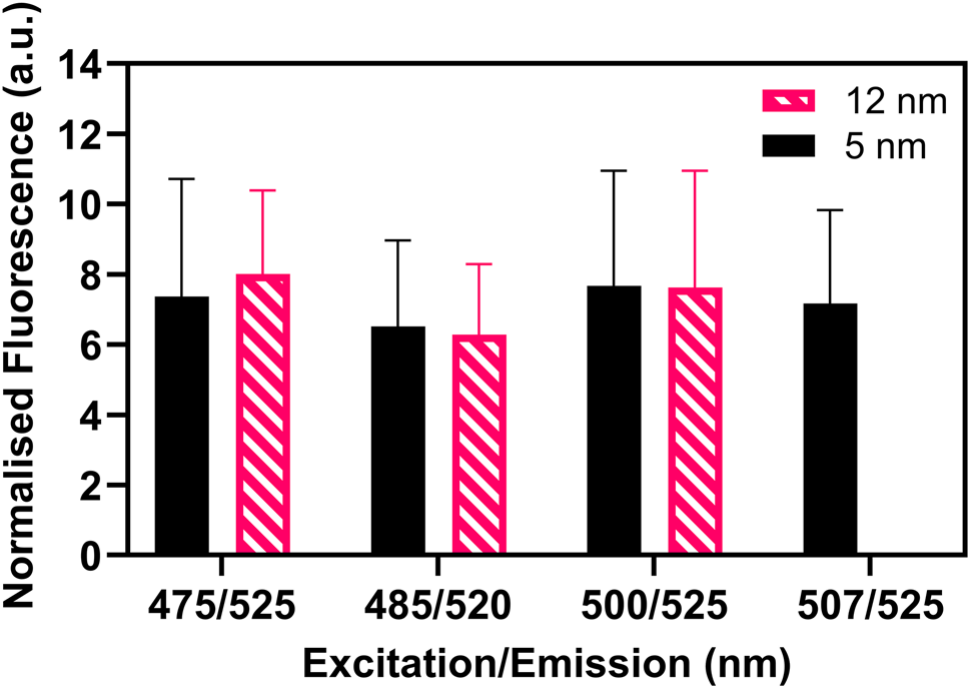
Normalised fluorescence of THP-1 cells incubated with CellROX Green for 1 h, captured at different excitation and emission wavelengths and emission bandwidths. Cells were treated with 100 µM of menadione for 30 min. Fluorescence at an excitation and emission of 507 nm and 525 nm, respectively, using an emission bandwidth of 12 nm was not measured because of instrument limitations. Error bars are based on the standard deviation of seven experimental replicates from two biological replicates (*n* = 7), with a single outlier removed from the following datasets: 475/525–12 nm, 485/520–5 nm, 12 nm 485/535. Samples showed no statistically significant differences according to Tukey’s post hoc testing (*p* > 0.05)

Parameters F and G in Table 1 are the excitation and emission combinations, and the emission bandwidths, respectively, explored in this study. Figure 5 shows the fluorescence of THP-1 cells treated with 100 µM of menadione, normalised to the untreated controls. Note, the fluorescence at 507/525 nm with a bandwidth of 12 nm was not measured because of instrument limitations. All samples showed similar normalised fluorescence and similar degrees of variance across the different emission bandwidths and excitation and emission wavelengths. This was also true for samples treated with 1, 5, 10, or 50 µM of menadione (Fig. S4). Differences in the average normalised fluorescence across these samples are likely a reflection of the inherent variance between biological replicates. These results highlight that any of the excitation/emission bandwidth combinations trialled are appropriate for the protocol utilising the Varioskan Lux microplate reader used in this study.

### Determining the importance of centrifugation prior to fluorescence measurements

A limitation of using plate reader-based fluorometry with suspension cells is that there can be large variation between replicates as the suspension cells are constantly in motion within the wells (Fan and Li 2014). Therefore, the number of cells that the detecting beam passes through may differ at a given time and between replicate samples (Fan and Li 2014). In an effort to minimise any effects, a centrifugation step was trialled before measuring fluorescence to concentrate the cells into a small pellet at the bottom of the wells.

**Fig. 6:**
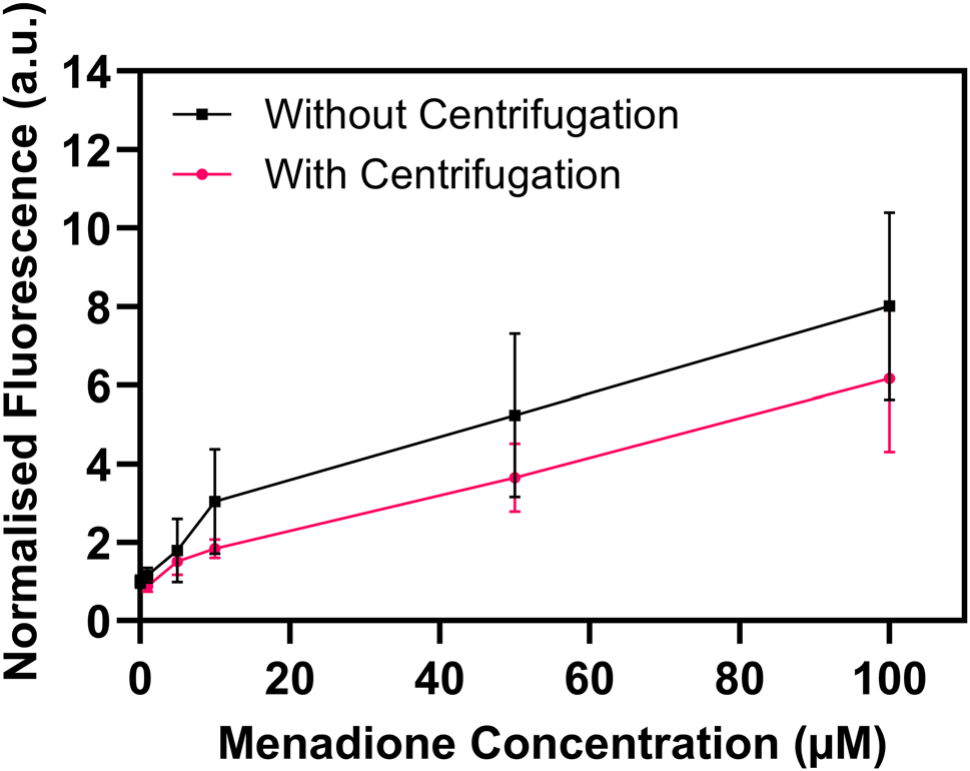
Normalised fluorescence of THP-1 cells incubated with CellROX Green for 1 h, with and without centrifugation, with fluorescence at an excitation and emission of 475 nm and 525 nm, respectively, using a 12-nm emission bandwidth. Cells were treated with 1 µM, 5 µM, 10 µM, 50 µM, or 100 µM menadione for 30 min. Error bars are based on the standard deviation of seven experimental replicates from two biological replicates (*n* = 7), with a single outlier removed from the following datasets: 100 µM–With Centrifugation, 100 µM–Without Centrifugation. No statistically significant differences were observed between samples with or without centrifugation according to Tukey’s post hoc testing (*p* > 0.05)

Figure 6 shows that the additional centrifugation step did not significantly impact fluorescence measurements, with the centrifuged and non-centrifuged samples being within error. This was also true at other investigated excitation and emission wavelengths (Fig. S5). This suggests that the centrifugation step is not necessary. However, most samples showed less variability across replicates when the centrifugation step was included. This highlights that, while not necessary, centrifugation can reduce replicate variability.

### Discussion and recommendations regarding plate reader-based protocol

**Fig. 7:**
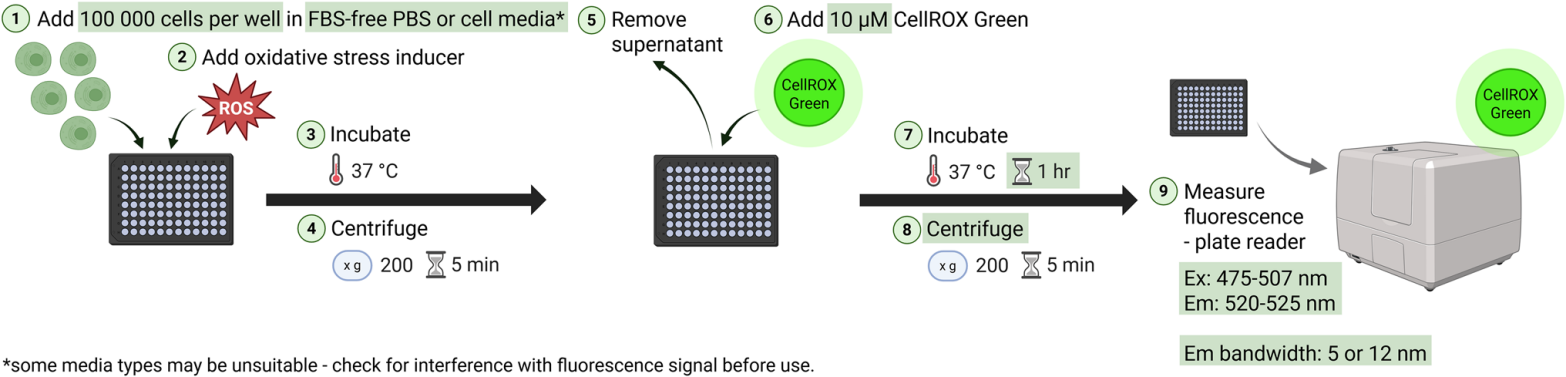
Schematic of optimised protocol for the measurement of intracellular ROS levels of suspended human monocyte leukemia (THP-1) cells. Green boxes identify the parameters optimised in this work. Note, steps 3–5 are not for experiments which do not require the addition of a compound. Foetal bovine serum (FBS), phosphate buffer saline (PBS), reactive oxygen species (ROS), excitation (Ex), emission (Em)

The developed plate reader-based protocol with the optimised parameters is visually represented in Fig. 7, and described below:

1. Add 100,000 cells in PBS in triplicate to a 96-well black clear-bottom plate.
2. Expose cells to the oxidative or experimental condition of interest. Include cell-free and untreated controls.
3. Incubate the plate at 37 °C.
4. Centrifuge the plate at 200 × *g* for 5 min to pellet the cells.
5. Remove as much of the supernatant as possible without disturbing the cell pellet and then resuspend the pellet in PBS.
6. Add CellROX Green to each sample to give a final CellROX Green concentration of 10 µM.
7. Incubate the plate for 1 h at 37 °C.
8. Centrifuge the plate at 200 × *g* for 5 min to pellet the cells. Do not remove the supernatant or disturb the cells.
9. Measure fluorescence at an excitation of 475 nm and emission of 500 nm to 600 nm to capture the quality of the sample. Depending on the sensitivity of the instrument, an excitation between 475 and 507 nm and an emission of between 520 and 525 nm is recommended. These excitation values can be modified depending on the emission peak position in cases where there is interference with test compounds.

Note, steps 3–5 are not required for experiments which do not require the addition of a compound (e.g. UV radiation).

This protocol has been optimised using THP-1 cells but has the potential to be used with other mammalian cell types. As with most protocols, parameters such as cell concentration, CellROX Green concentration, and incubation time may require further optimisation for other cell types and other treatments depending on the amount of ROS generated. While PBS is suggested for this protocol, phenol-free, FBS-free media may also be appropriate but should be empirically tested for its suitability before use. Though not necessary, centrifugation before obtaining fluorescence is advised to reduce replicate variability.

Using the Varioskan Lux microplate reader, all investigated excitation and emission combinations with the appropriate emission bandwidth were shown to be appropriate for this protocol. These findings are supported by existing literature on CellROX Green, with McBee et al. (2017) using an excitation filter and emission filter of 485/20 nm and 528/20 nm, respectively, for capturing fluorescence of bacterial samples, and Zwicker et al. (2022) using an excitation and emission of 490 nm and 535 nm, respectively, for capturing the fluorescence of cell lysate samples. Though McBee et al. (2017) and Zwicker et al. (2022) used slightly higher emission wavelengths, our data suggested that at an excitation of 475 nm, fluorescence of experimental samples peaked between 522 and 532 nm (Fig. S6). It is our suggestion that at an excitation of 475 nm, an emission spectrum should be obtained between 500 to 600 nm. Changes in the peak position, the relative noisiness of the data, and/or unusual spikes in the data across these wavelengths provide insights into the quality of the samples or provide evidence of possible interference by treatment compounds which may impact the fluorescence data. Thus, it is our suggestion that an emission spectral scan should be obtained at the start of the investigation to select the most appropriate single emission wavelength for the sample or treatment type, followed by a single emission wavelength readout for the intended screening application.

A limitation of using some commercial kits and probes such as CellROX Green is that their chemical reactivity and structure are not disclosed. This can lead to challenges in interpreting results and identifying method-specific artefacts (Murphy et al. 2022). However, this challenge extends to ROS probes with known structures such as the DCFDA probe which also possesses notable susceptibility to method-specific artefacts among other limitations (Murphy et al. 2022). Despite these limitations, non-specific ROS probes have significant value for initial assessment of oxidative stress (Murphy et al. 2022). ROS-specific methods of investigation such as genetically encoded fluorescent protein sensors and liquid chromatography–mass spectrometry can then be used for more in-depth investigations (Murphy et al. 2022).

### Comparing the plate reader-based protocol with flow cytometry

**Fig. 8:**
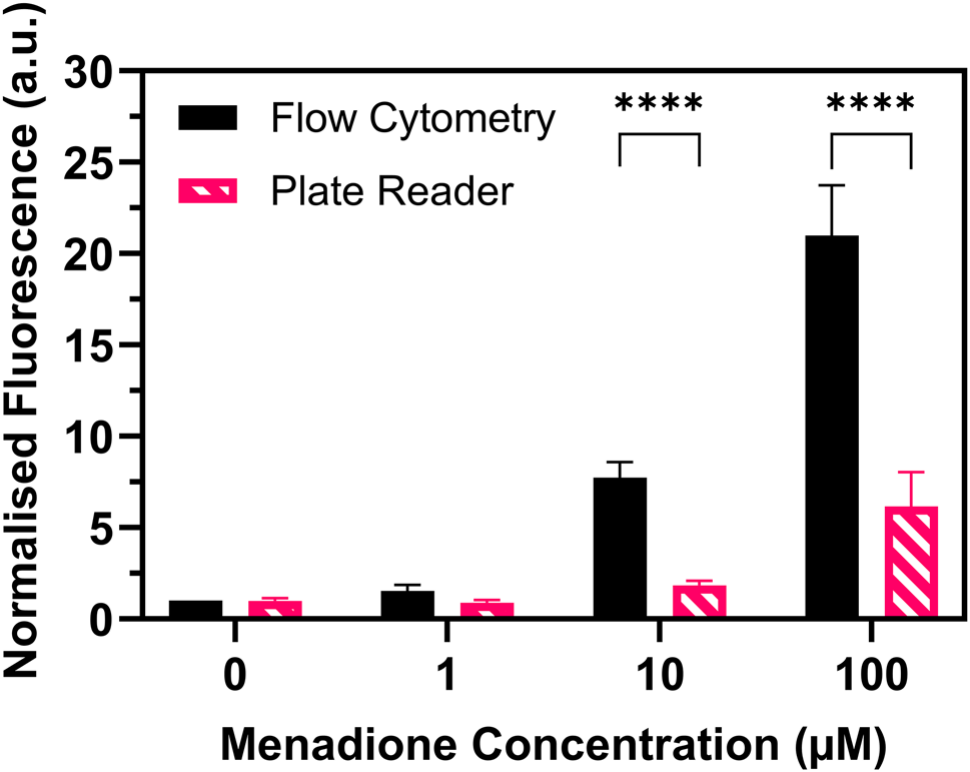
Normalised fluorescence of THP-1 cells treated with 1 µM, 10 µM, or 100 µM of menadione for 30 min as captured using flow cytometry and microplate-based fluorometry (plate reader). For flow cytometry, errors are based on the standard deviation of duplicate experimental replicates. For each experimental replicate, 10,000 events were captured. The forward scatter threshold was adjusted to two million and a forward scatter versus side scatter density plot of the untreated sample was used to gate out cellular debris from analysis for all samples. For the plate reader data, fluorescence was measured at an excitation of 475 nm and emission of 525 nm, and error bars are based on the standard deviation of seven experimental replicates from two biological replicates (*n* = 7), with a single outlier removed from the 100 µM plate reader dataset. Notable differences as determined by Tukey’s post hoc testing are shown as follows: ****p ≤ 0.0001

CellROX Green has traditionally been paired with either flow cytometry or fluorescence microscopy to measure intracellular ROS levels of cells. Figure 8 shows the normalised fluorescence of THP-1 cells after treatment with menadione as captured using either the developed plate reader-based protocol or flow cytometry. Flow cytometry yielded higher normalised fluorescence than the plate reader-based protocol, indicating greater sensitivity. The significant difference in the sensitivities of these methods was expected as the flow cytometer is well recognised within the literature as a more sensitive instrument than a plate reader for fluorescence-based intracellular ROS measurements (Djiadeu et al. 2017; Ruijter et al. 2024). As a result of the nature of the methods, the flow cytometry required slightly different CellROX Green concentrations and used different wavelengths than the plate reader protocol. Therefore, the comparison is best considered a benchmarking exercise rather than a direct analytical validation. However, despite this, both assays recorded an approximate threefold increase in normalised fluorescence for the 100 µM menadione samples as compared to the 10 µM samples. This highlights that, though flow cytometry is more sensitive than the plate reader, the results obtained from the plate reader protocol are comparable to those obtained via flow cytometry if high enough ROS is generated (as was the case with the 10 µM and 100 µM menadione treatments). When comparing the results obtained via plate reader-based fluorometry and flow cytometry using the DCFDA probe, Ruijter et al. (2024) found that the results correlated poorly. This may suggest that the intracellular ROS induced by the selected treatments were not at a high enough level to be accurately detected by the plate reader, or that the DCFDA probe does not yield comparable results to the flow cytometer when using a plate reader. This is in contrast to the plate reader-based protocol presented here which gave comparable results to the flow cytometer at high treatment concentrations (of 10 µM and 100 µM menadione).

Flow cytometry was shown to be more sensitive than the plate reader protocol for the measurement of intracellular ROS levels of the treated THP-1 cells. However, there are several advantages of using the developed plate reader-based protocol which makes it more suitable for screening applications which require only an approximate measure of oxidative stress levels. Firstly, the use of the microplate reader allows for the fluorescence of 96 samples to be measured at a single wavelength in under a minute. In contrast, it took up to 5 min to measure one sample using the flow cytometer and therefore would take approximately 8 h to measure 96 samples. Secondly, the plate reader-based protocol used a final volume of 70 µL per well to measure fluorescence of the samples, while a much greater volume of 1.5 mL was used for flow cytometry. Finally, microplate readers with fluorescence detection are more common in labs than flow cytometers, presumably because of their lower cost and more versatile use. Therefore, for applications which require a general indication of oxidative stress levels using a scalable, simple, and quick method, the developed plate reader-based protocol has significant advantages over flow cytometry.

### Validating the plate reader-based protocol with suspended HaCaT cells

**Fig. 9:**
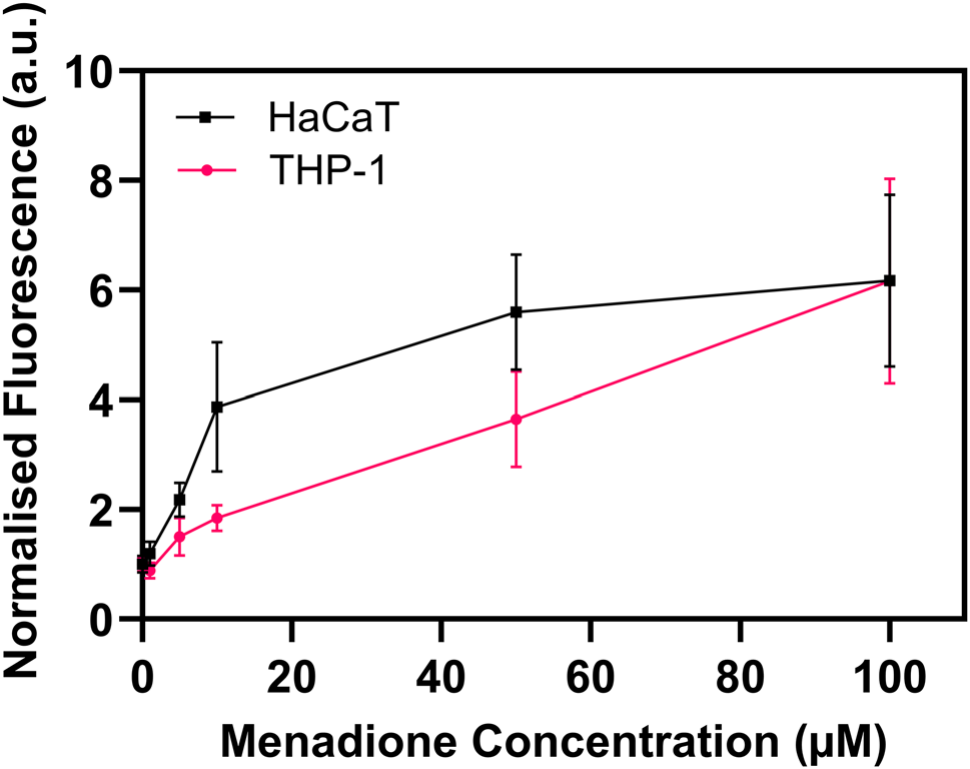
Normalised fluorescence of suspended HaCaT and THP-1 cells treated with 1 µM, 5 µM, 10 µM, 50 µM, or 100 μM of menadione for 30 min. Samples were incubated with 10 µM of CellROX Green for 1 h. Fluorescence was measured at an excitation of 475 nm and emission of 525 nm. Error bars are based on the standard deviation of seven experimental replicates from two biological replicates (*n* = 7), with a single outlier removed from the following datasets: 100 µM–THP-1, 10 µM–HaCaT. No statistically significant differences were observed between samples according to Tukey’s post hoc testing (*p* > 0.05)

To demonstrate that the plate reader-based protocol could be used with other cell lines, the ROS levels of menadione-treated suspended HaCaT cells were assessed (using the protocol conditions as listed in Table 1). As shown in Fig. 9 and similar to the THP-1 cells, the plate reader-based protocol was able to capture changes in the relative fluorescence and therefore ROS levels of HaCaT cells after treatment with up to 100 µM of menadione. Treatment with 10 µM and 50 µM of menadione resulted in higher normalised fluorescence in the HaCaT cells compared to the THP-1 cells, indicative of relatively greater oxidative stress to the HaCaT cells. HaCaT cells are an adherent cell line and therefore when in their suspended form are likely more intrinsically stressed and/or more susceptible to stress-inducing compounds such as menadione. Treatment with 100 µM of menadione resulted in near identical fluorescence for THP-1 and HaCaT cells, suggesting similar levels of ROS triggered at this concentration. The data in Fig. 9 highlights that this plate reader-based protocol, developed for THP-1 cells, can also be applied to HaCaT cells. This suggests that future applications to other cell types should be possible, although cell-specific validation would be required.

## Conclusion

The accurate measurement of intracellular ROS levels provides critical insights into cell health. However, as a result of the low quality, high reactivity, and short half-life of ROS, plate reader-based fluorimetry methods of measuring intracellular ROS levels in mammalian cells are scarce. To the best of our knowledge, our team is the first to have developed a semi-quantitative, plate reader-based protocol using CellROX Green that can be used to measure the intracellular ROS levels of viable THP-1 cells, a model suspension mammalian cell line.

With menadione being the oxidative stress inducer, a cell concentration of 100,000 cells per well, a CellROX Green concentration of 10 µM, a CellROX Green incubation time of 1 h, and the inclusion of a centrifugation step before obtaining fluorescence were shown to provide the most accurate measurement of intracellular ROS levels in THP-1 cells. For capturing fluorescence at a single emission wavelength utilising the specific conditions and ranges of the Varioskan Lux microplate reader, an excitation between 475 and 507 nm and an emission between 520 and 532 nm were shown to provide accurate data representative of general ROS levels within THP-1 cells. While it is likely that similar ranges will work for other plate readers, some instrument-specific optimisation of the protocol may be necessary. Nonetheless, the developed protocol could also be adapted for adherent mammalian cells, but larger wells would be necessary to support the cell concentrations required.

As expected, the plate reader protocol was shown to be less sensitive than the flow cytometer. However, the trends are consistent between the two methods. These findings confirm that the developed plate reader protocol can be used as a quick and simple method for detecting moderate to high levels of ROS within suspended cells, and to study changes in oxidative stress. If ROS levels are expected to be relatively small, it is possible that the more sensitive flow cytometer method will be required.

Unlike the DCFDA and DHR123 probes, a limitation of using CellROX Green is that its chemical structure is not available and therefore an understanding of the probe’s properties and how this may affect the accuracy of the fluorescence measurements is not possible. Thus, while the one-step mechanism of CellROX Green may eliminate susceptibility to self-amplification compared to the DCFDA and DHR123 probes, this cannot be concluded without knowledge of the chemical structure of the probe. Nonetheless, the developed protocol can be considered a reliable alternative to the DCFDA and DHR123 plate reader-based protocols.

Unique to the protocol presented in this work is the elimination of the washing step prior to measuring fluorescence. Unlike the DCFDA and DHR123 plate reader protocols, this work showed that the unbound CellROX Green did not significantly contribute to background fluorescence. Therefore, the presented protocol does not require removal of the unbound probe, thus minimising cell loss and making it more appropriate for suspension cells than the existing DCFDA and DHR123 plate reader-based protocols. Unlike other published CellROX Green protocols (McBee et al. 2017; Zwicker et al. 2022), the developed protocol can provide insights into the general ROS levels of intact and viable THP-1 cells, facilitating real-time measurement of ROS levels. Further, the use of a benchtop plate reader instead of a fluorescence microscope or flow cytometer increases the accessibility of CellROX Green. Ultimately, our protocol provides a fast, scalable, and user-friendly method of identifying moderate to large differences in oxidative stress levels of suspended THP-1 cells which makes it ideal for screening applications, with the potential to have applicability across other suspended mammalian cell types.

## Supplementary Information

Below is the link to the electronic supplementary material.

Supplementary file1 (PDF 469 KB)

## Data Availability

All data supporting the findings of this study are available within the paper and its Supplementary Information. Raw data can be requested from the authors.
