# Supplementary material for: A high-throughput, plate reader-based method for the assessment of oxidative stress in suspension mammalian cells using CellROX Green
Source: Histochem Cell Biol. 2026 Jul 27;164(1):63. doi: 10.1007/s00418-026-02517-2 (PMC13407569; doi:10.1007/s00418-026-02517-2)
Supplement: Supplementary file 1 — Supplementary file1 (PDF 469 KB) [file 418_2026_2517_MOESM1_ESM.pdf]

Supplementary

A high-throughput, plate reader-based method for the assessment of oxidative stress in suspension mammalian cells using CellROX Green

Histochemistry and Cell Biology

Miyah N. Awad,[1] Amanda N. Abraham,[1] Philipp Reineck,[1] Sara Pourshahrestani,[1] Izabela Milogrodzka,[1] Aaron Elbourne,[1] Tamar L. Greaves,[1] Gary Bryant,[1] and Saffron J. Bryant.[1]*

[1]School of Science, STEM College, RMIT University, Melbourne, VIC 3001, Australia.

*saffron.bryant@rmit.edu.au

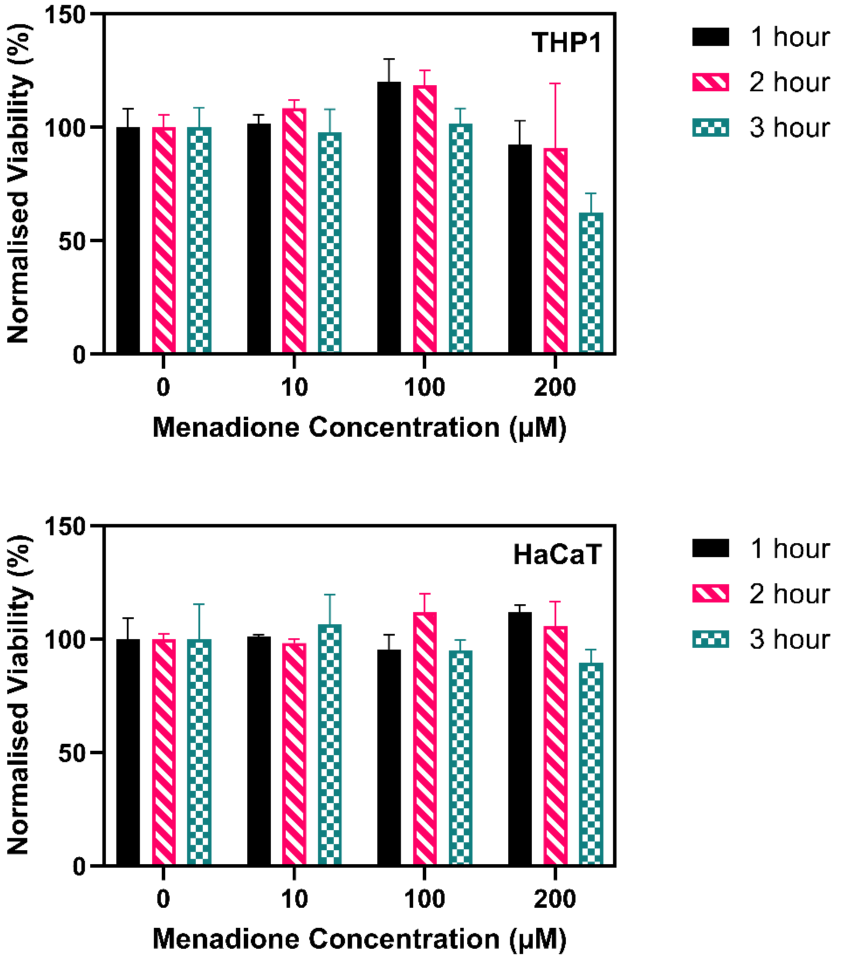

Fig S1 Normalised viability of THP-1 and HaCaT cells after 1, 2, and 3 hours post-menadione treatment for 30 minutes. Viability is normalised to the untreated control. Error bars are based on the standard deviation of two experimental replicates. For both THP-1 and HaCaT cells, treatment with 10 µM and 100 µM menadione resulted in no significant change to viability 3 hours post-treatment. In contrast, treatment with 200 µM of menadione resulted in a reduction in viability 3 hours post-treatment.

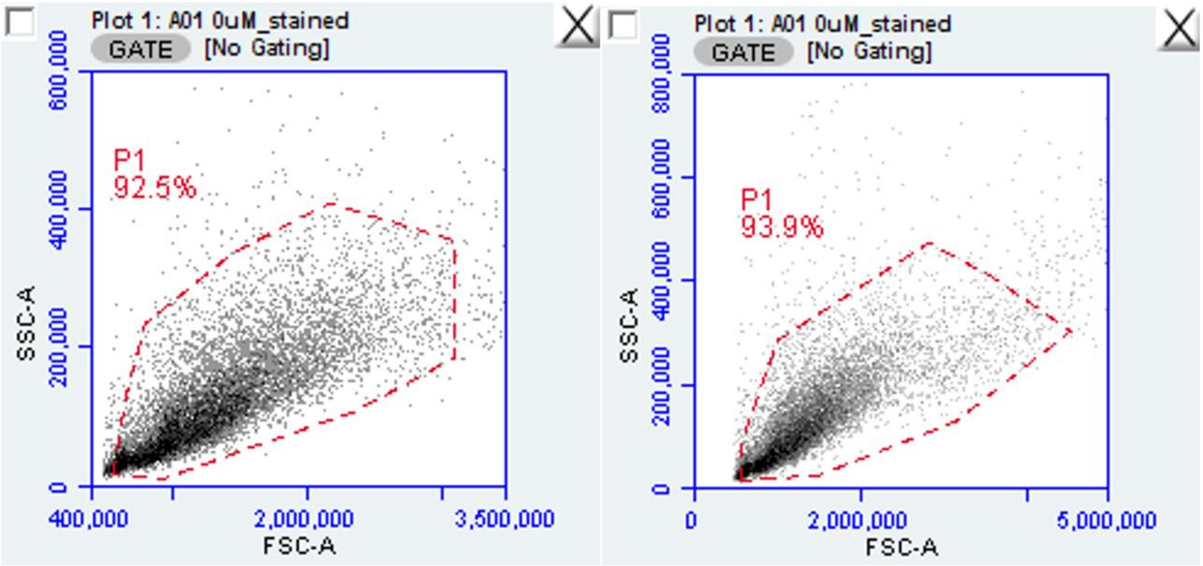

Fig S2 Gating of stained untreated samples used for flow cytometry experiments. The forward scatter threshold was adjusted to two million and a forward scatter versus side scatter density plot of the untreated sample was used to gate out cellular debris from analysis. This gating was used to gate all other samples belonging to that biological replicate.

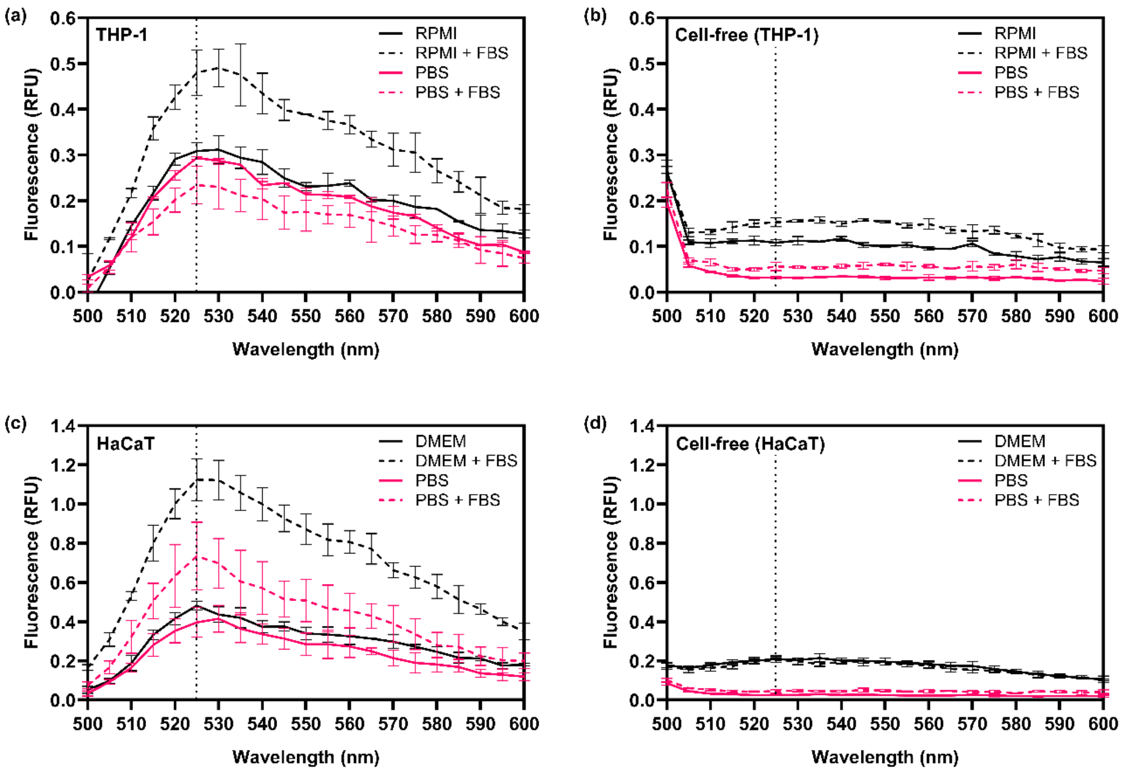

Fig S3 Fluorescence at an excitation of 475 nm of (a) THP-1 cell samples, (b) and cell-free controls, and (c) HaCaT cell samples, and (d) cell-free controls – PBS, PBS supplemented with FBS, RPMI, RPMI supplemented with FBS, DMEM, DMEM supplemented with FBS. For the cell samples, fluorescence is reported after the fluorescence of the untreated cell controls was subtracted. For the cell-free controls, raw fluorescence is plotted. Dotted line shows peak emission at 525 nm. Error bars are based on the standard deviation of at least two experimental replicates.

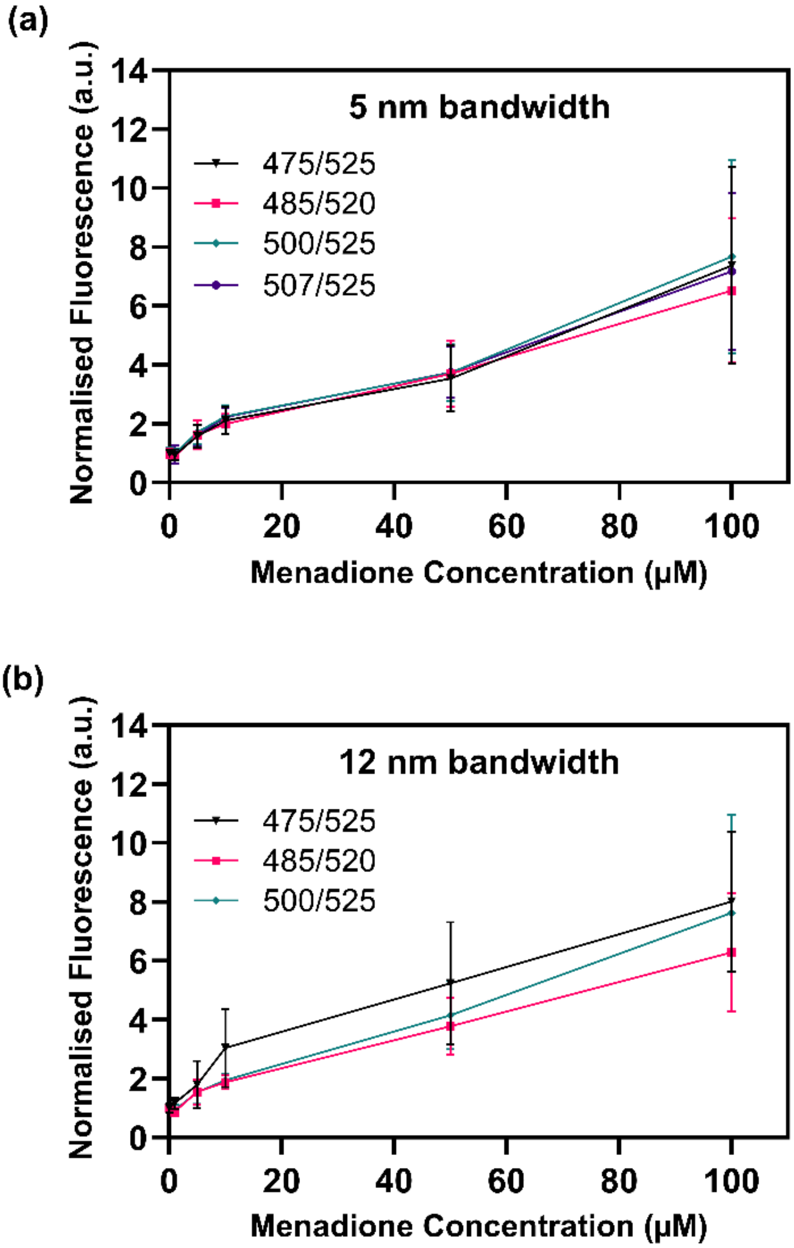

Fig S4 Normalised fluorescence of THP-1 cells incubated with CellROX Green for 1 hour, captured at different excitation and emission wavelengths and emission bandwidths. Cells were treated with 1 µM, 5 µM, 10 µM, 50 µM, or 100 µM menadione for 30 minutes. Fluorescence at an excitation and emission of 507 nm and 525 nm, respectively using an emission bandwidth of 12 nm was not measured due to instrument limitations. Error bars are based on the standard deviation of at least two experimental replicates.

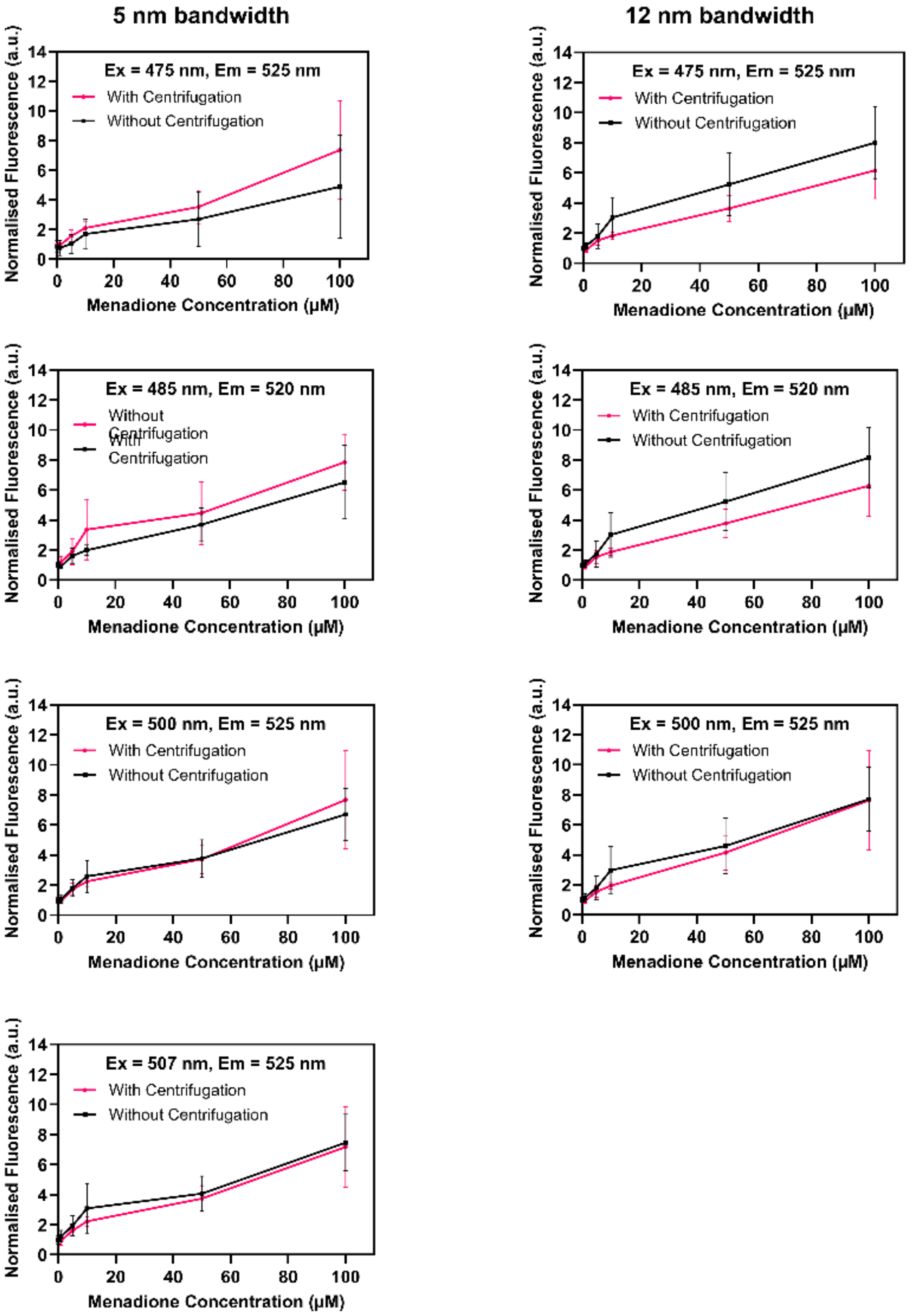

Fig S5 Normalised fluorescence of THP-1 cells incubated with CellROX Green for 1 hour, captured at different excitation and emission wavelengths and emission bandwidths. Cells were treated with 1 µM, 5 µM, 10 µM, 50 µM, or 100 µM menadione for 30 minutes. Fluorescence was captured before and after centrifugation. Fluorescence was normalised to the untreated control. Error bars are based on the standard deviation of at least two experimental replicates.

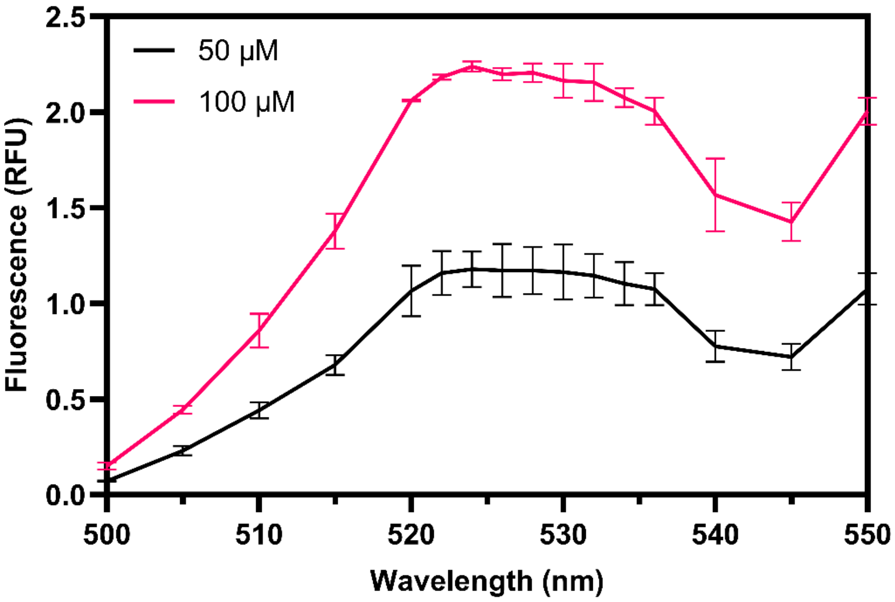

Fig S6 Fluorescence peak of THP-1 cells incubated with CellROX Green for 1 hour, captured at an excitation of 475 nm. Cells were treated with 50 μM or 100 μM of menadione for 30 minutes. Fluorescence was captured after centrifugation. Fluorescence of the cell-free control was subtracted from the cell samples. Error bars are based on the standard deviation of two experimental replicates. Fluorescence peaks between 522 nm and 532 nm using an excitation of 475 nm.
